# Characterizing tramadol users with potentially inappropriate co-medications: A latent class analysis among older adults

**DOI:** 10.1371/journal.pone.0246426

**Published:** 2021-02-19

**Authors:** Bo Ram Yang, Hye-Yeon Um, Min Taek Lee, Myo Song Kim, Sun-Young Jung

**Affiliations:** 1 College of Pharmacy, Chungnam National University, Daejeon, Republic of Korea; 2 Department of Statistics, Ewha Womans University, Seoul, Republic of Korea; 3 College of Pharmacy, Chung-Ang University, Seoul, Republic of Korea; 4 Department of Global Innovative Drugs, Graduate School of Chung-Ang University, Seoul, Republic of Korea; University of Tennessee Health Science Center, UNITED STATES

## Abstract

**Background:**

Although tramadol is an effective weak opioid analgesic, careful monitoring of potential central nervous system adverse reactions in older adults is needed, especially when used with concomitant medications which may trigger the adverse effects. We aimed to characterize tramadol users with potentially inappropriate co-medications in older adults using a latent class analysis (LCA).

**Method:**

Patients aged 65 years or older using tramadol and receiving potentially inappropriate co-medications were included from a nationwide healthcare claims database. We defined antidepressants, first-generation antihistamines, and anxiolytics as potentially inappropriate co-medications. We applied an LCA for grouping tramadol users based on the common characteristics of medication use and healthcare utilization, and each patient was probabilistically assigned to a class. Patients’ characteristics in different latent classes were compared. Potential adverse drug reactions (ADRs) was defined as the any visits for emergency department after the occurrence of potentially inappropriate co-medications. Logistic regression analysis was used to examine the association between latent classes and potential ADRs.

**Results:**

We identified four distinct latent classes of tramadol users representing different patterns of co-medications: multiple potential drug-drug interaction (pDDI) combination users, antihistamines-tramadol users, antidepressants-tramadol users, and anxiolytics-tramadol users. Multiple pDDI combination users showed high proportion of regular tramadol use, tended to visit more medical institutions, and had a high Charlson comorbidity score. The duration of use of potentially inappropriate co-medications with tramadol was the longest in multiple pDDI combination users and the shortest in antihistamines-tramadol users.

When compared with antihistamines-tramadol users, increased potential ADR risk was observed in multiple pDDI combination users (adjusted odds ratio (OR), 1.81; 95% confidence interval (CI), 1.75–1.88), antidepressants-tramadol users (1.24; 1.19–1.29), and anxiolytics-tramadol users (1.04; 1.00–1.08).

**Conclusions:**

Four distinct classes were identified among older adults using tramadol and potentially inappropriate co-medications. Differences in potential ADR risk were observed between these classes. These findings may help to identify patients at a high risk for ADRs owing to potentially inappropriate co-medications with tramadol.

## Introduction

Tramadol is one of the most commonly used opioid analgesics worldwide [1, 2]. In older adults, while pain-related conditions become more prevalent [3], age-related physiological changes increase the vulnerability to the adverse effects of commonly used analgesics [4]. Although concerns about gastrointestinal bleeding and renal insufficiency risks associated with non-steroidal anti-inflammatory drugs (NSAIDs) [5] might favour tramadol as a safe alternative [6], Beers criteria recommend that tramadol should be avoided or used with caution since it is potentially inappropriate in older adults owing to the risk of central nervous system (CNS) adverse effects [7].

Besides a weak agonistic effect on the μ-opioid receptors, tramadol acts by simultaneously inhibiting norepinephrine and serotonin reuptake [8]. This unique mechanism was associated with a dose-related increase in adverse drug reaction (ADR) risk of tramadol, including serotonin syndrome (SS), seizures, and sedation [7, 9–11]. Because older adults with chronic pain syndromes are prone to have multiple comorbidities, adverse reactions may be triggered by potential interactions with commonly used co-medications [12].

Concomitant use of selective serotonin reuptake inhibitors (SSRIs), benzodiazepines (BZDs), or first-generation antihistamines with tramadol may increase SS risk [13–16]. In addition, the concomitant use of first-generation antihistamines with tramadol may increase the anticholinergic and sedative effects [10]. Several cases of SS owing to the concomitant use of SSRIs, BZDs, or first-generation antihistamines with tramadol have been reported [13–16]. Cases of excess anticholinergic or sedative effects associated with the concomitant use of first-generation antihistamines with tramadol also have been reported [10]. Cases of fatal CNS depression owing to possible interaction between tramadol and BZDs also exist [17].

Despite these potential harms resulting from drug interactions with antidepressants, anxiolytics (including BZDs and non-BZDs), and first-generation antihistamines in tramadol users, population-based studies on patterns of using the medications have been lacking. A population-based assessment of the patterns of tramadol users with potentially inappropriate co-medications and the associated factors is important to establish public strategies to prevent safety problems in older adults. Because of the diverse underlying diseases and healthcare utilization patterns, different drug-drug interactions (DDIs) can occur depending on the frequently used co-medications including antidepressants, anxiolytics, and first-generation antihistamines. Therefore, in this study, we aimed to identify groups of tramadol users with distinct co-medication profiles using a latent class analysis (LCA), a statistical method for grouping individuals sharing common characteristics into distinct ‘clusters’ [18].

The objective of this study was to identify latent classes of tramadol users, which reflected the real-world older population, with potentially inappropriate co-medications, using multiple indicators (including medication use and healthcare utilization) recorded in a nationwide claims database for older adults so as to provide an insight into the characteristics of homogenous groups of patients. Additionally, we assessed the differences in patients’ and providers’ characteristics and the occurrence of potential ADRs among the different classes of tramadol users to identify the patients who required intervention to reduce the risk of potential ADRs.

## Materials and methods

### Data source

We extracted data on elderly patients from the 2016 update of Korea Health Insurance Review and Assessment Service-Adult Patient Sample (HIRA-APS), which is a public database [19]. The HIRA-APS database is constructed by a stratified random sampling method for age intervals of 5 years and sex of elderly patients ≥ 65 years. It includes claims data of approximately 1 million elderly patients, which accounts for 20% of the elderly population in Korea and contains each patient’s unique encrypted identification number, age, sex, prescription number, prescription drugs (generic name, prescription date, supply days, dose) and medical institution identifier. Information on outpatient, inpatient, and ED visits are also included in the HIRA-APS database. The diagnosis was coded according to the International Classification of Disease, Tenth Revision (ICD-10). Because we used the anonymized database, we could not access any identifying information.

The study protocol was exempted from review by the Institutional Review Board of Chung-Ang University (IRB number: 1041078-201707-HR-137-01).

### Study population and study drugs

We performed a population-based cross-sectional study. The study population included all patients aged ≥ 65 years, who received at least one potentially inappropriate co-medications with tramadol in 2016. Patients diagnosed with cancer were excluded to focus on patients with non-cancer pain. Presence of potentially inappropriate co-medications with tramadol were defined as overlapping between the prescription periods of tramadol and potentially inappropriate co-medications for at least one day based on each drug prescription date and duration. Potentially inappropriate co-medications with tramadol were classified into three groups: 1) antidepressants, including SSRIs, tricyclic antidepressants (TCAs), and monoamine oxidase inhibitors (MAOIs), 2) first-generation antihistamines, and 3) anxiolytics, including BZDs and non-BZDs. Detailed drug list is presented in S1 Table.

### Assessment of potentially inappropriate co-medications with tramadol

In order to describe potential clusters of tramadol users with potentially inappropriate co-medications, we assessed factors that may related to co-medication patterns were identified according to concomitant use of each medication (antihistamines, anxiolytics, and antidepressants), duration of co-medication, and duration of tramadol use in study period using medication possession ratio (MPR) to classify the patients having potentially inappropriate co-medications with tramadol. In addition, we also assessed factors associated healthcare settings including switching healthcare institutions, and prescription made in primary care clinics. Seven categorical variables for potentially inappropriate co-medications included type of each co-medication, regular use of tramadol, longer concomitant use, prescription at clinics, and healthcare switching. Regular use of tramadol was assessed using the MPR, defined as the ratio of the sum of days with medication supply to the total number of days in a defined period, and patients having an MPR ≥ 0.6 were considered regular users. Duration of concomitant use was calculated as the sum of overlapping periods of potentially inappropriate co-medications. If the duration of overlapped use between tramadol and potentially inappropriate co-medications more than 10 days, it was considered long concomitant use. Several cut offs values for long concomitant use (concomitant use more than 10 days, 20 days, and 30 days) and regular tramadol use (MPR ≥ 0.6, MPR ≥ 0.7, and MPR ≥ 0.8) were considered based on the distribution of duration of concomitant use and tramadol use in our database and literature review [20, 21]. We selected the cut off value by class distinguishability after conducting several exploratory analyses.

Healthcare switching was defined as patients receiving tramadol prescriptions from more than two different medical institutions. Prescription at clinics referred to patients receiving tramadol at clinics at least once.

### Latent class analysis

Using the seven variables described above, the LCA method was used to assign each patient to a ‘class.’ Among the traditional clustering methods which measure the distance from randomly selected observations, LCA is a model-based clustering method which calculates the probability that an observation will be a member of certain latent classes, based on maximum likelihood estimation. Each participant is assigned to the group which has the highest probability of having that participant [22]. This statistical method posits latent classes based on underlying patterns that cannot be directly observed [18]. The objective of LCA is to find the smallest number of groups that best describes the associations.

We fitted 2-class to 6-class models to identify the optimum number of classes. The model fit indices, including the likelihood ratio G2 statistic, Akaike information criterion, and Bayesian information criterion, were evaluated. We also considered model interpretability, each class distinguishability, and subgroup size triviality, such that no class had a near-zero probability of membership [23].

We selected the best-fit model by estimating the prevalence of patient membership in the latent classes. To examine whether these results were robust, sensitivity analysis by sex and hospitalization was conducted.

### Definition of potential ADRs

We assessed the occurrence of potential ADRs after receiving potentially inappropriate co-medications and evaluated whether the occurrence of potential ADRs differed in the four latent classes. We defined potential ADRs as any visit to an emergency department (ED) after the first occurrence of potentially inappropriate co-medications with tramadol.

### Statistical analysis

A descriptive analysis of demographics (age, sex, and type of insurance), comorbidities (myocardial infarction, congestive heart failure, cerebrovascular disease, liver disease, and renal disease), medication use (MPR of tramadol and duration of concomitant pDDI), healthcare utilization (number of visited institutions and type of institutions), geographic region (Seoul, urban area, and rural area), and provider specialty (internal medicine, neurology, general surgery, psychiatry, neurosurgery, rehabilitation, and family medicine) was conducted for the four latent classes. The type of institution was defined according to the healthcare institution that mostly prescribed tramadol for each patient in 2016, and number of visited institutions was defined as the number of healthcare institutions that prescribed tramadol. We captured information on demographics, geographic region, and provider specialty for the first prescription of tramadol; medication use, comorbidities, and healthcare utilization were identified using both inpatient data and outpatient visits from January 1, 2016 to December 31, 2016.

We calculated the means, standard deviations (SD), and proportions in each latent class. Chi-squared test was used to compare the proportions of categorical variables, whereas analysis of variance (ANOVA) was used to compare continuous variables among different latent classes. We also assessed the occurrence of potential ADRs in each latent class. Crude odds ratios (ORs) and 95% CIs were calculated using univariable logistic regression. The reference category was antihistamines-tramadol users. A multivariable logistic regression analysis was performed to calculate the adjusted ORs and 95% CIs for estimating the association between each latent class and potential ADR with adjustment for age, sex, type of insurance, comorbidities (myocardial infarction, congestive heart failure, cerebrovascular disease, liver disease, renal disease), and geographic regions.

All statistical tests were performed 2-sided and results with p values of less than 0.05 were considered statistically significant. SAS version 9.4 (SAS Institute, Inc., Cary, NC, USA) was used to construct the database, and R-project 3.4.1 was used to perform the LCA.

## Results

**Fig 1:**
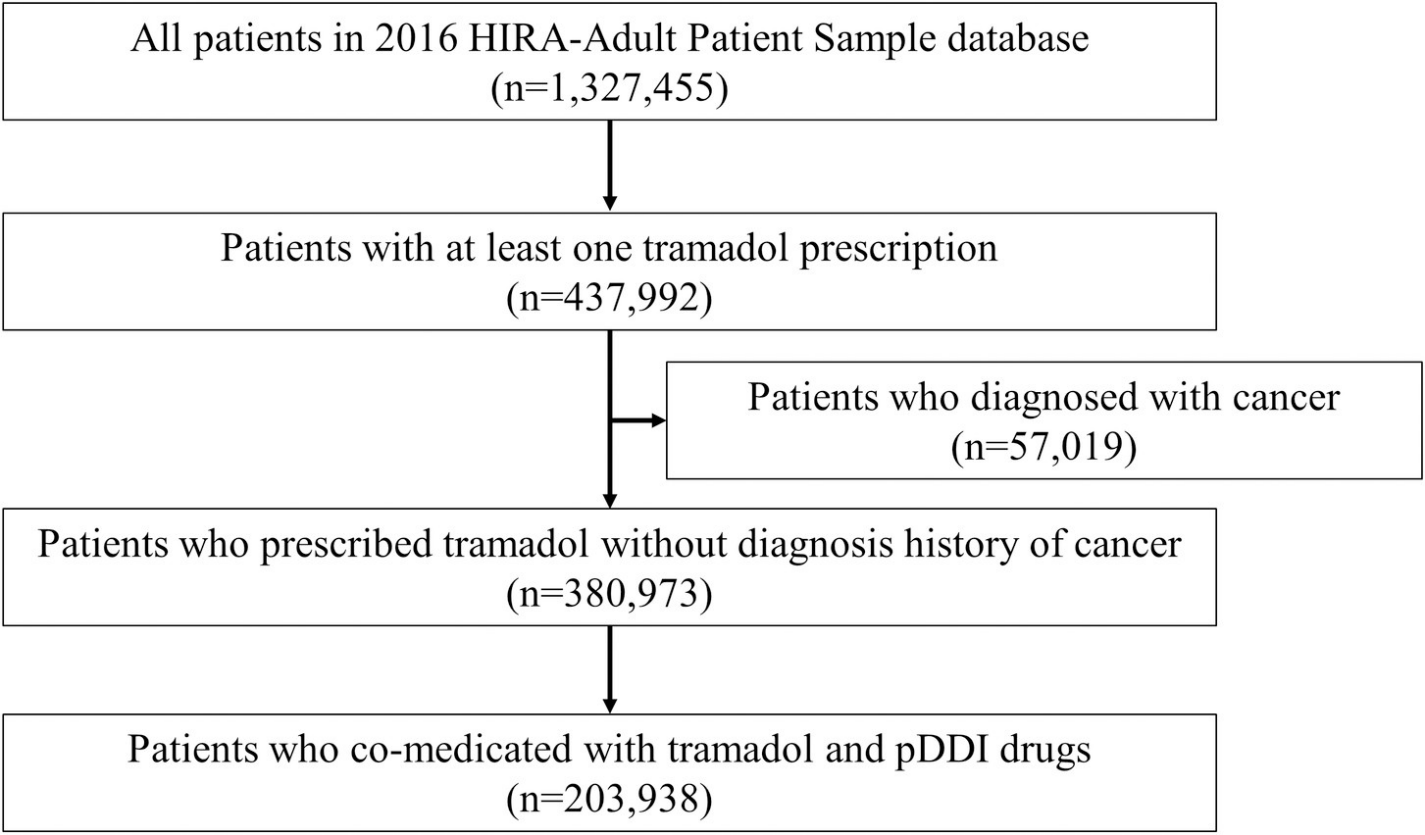
Selection of study participants from 2016 HIRA-adult patient sample database. pDDI, potential drug-drug interaction.

We identified 203,938 patients who had at least one potentially inappropriate co-medications with tramadol in 2016 (Fig 1).

**Fig 2:**
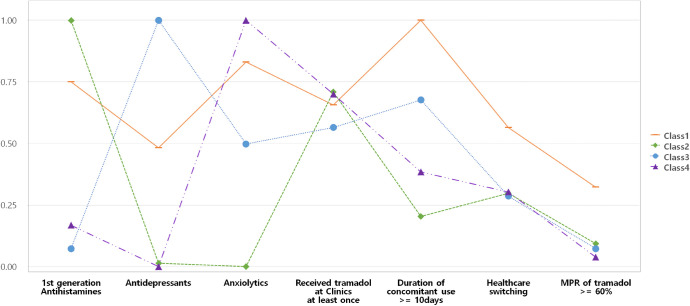
Class probability in the four latent classes of tramadol users with potential drug-drug interactions. *Class 1, multiple pDDI combination users; Class 2, antihistamines-tramadol users; Class 3, antidepressants-tramadol users; and Class 4, anxiolytics-tramadol users. MPR, medication possession ratio.

The study population was categorized into four latent classes based on the model fit data and model interpretability (S2 Table). The estimated probability of the seven variables included in the LCA is shown in Fig 2. A label was assigned to each class based on the conditional item response probabilities. Four classes, multiple potential drug-drug interaction (pDDI) combination users (Class 1), first-generation antihistamines-tramadol users (Class 2), antidepressants-tramadol users (Class 3), and anxiolytics-tramadol users (Class 4) were distinguished.

Among multiple pDDI combination users, 86.2% received anxiolytics, followed by first-generation antihistamines (81.5%), and antidepressants (51%). All patients among antihistamines-tramadol, antidepressants-tramadol, and anxiolytics-tramadol users were prescribed the labelled interacting drug. Compared with other classes, multiple pDDI combination users exhibited high proportion of regular tramadol users and tended to visit more than one medical institution for tramadol prescription. The duration of concomitant use was relatively short in antihistamines-tramadol users and long in multiple pDDI combination users (Table 1). Results of the sensitivity analysis showed that the latent class model had almost the same classes if stratified by sex (S1 Fig, S3 Table).

**Table 1:** Characteristics of the four latent classes of tramadol users with potential drug-drug interactions.

	Class 1	Class 2	Class 3	Class 4
	(Multiple pDDI combination users)	(Antihistamines-tramadol users)	(Antidepressants-tramadol users)	(Anxiolytics-tramadol users)
Total number of patients	63,827	50,681	33,113	56,317
Class probability (Prevalence)	0.313	0.249	0.162	0.276
**Received tramadol at clinic at least once**	42,701 (66.9)	36,519 (72.1)	17,223 (52)	39,220 (69.6)
**MPR of tramadol ≥ 60%**	21,796 (34.1)	3,987 (7.9)	1,617 (4.9)	1,096 (1.9)
**1^st^ generation antihistamines**	52,033 (81.5)	50,681 (100)	598 (1.8)	5,509 (9.8)
**Antidepressants**	32,525 (51)	633 (1.2)	33,113 (100)	-
**Anxiolytics**	55,010 (86.2)	-	16,089 (48.6)	56,317 (100)
**Healthcare switching^a^**	35,948 (56.3)	15,573 (30.7)	7,901 (23.9)	18,421 (32.7)
**Duration of concomitant use ≥ 10 days^b^**	63,827 (100)	12,141 (24)	22,110 (66.7)	21,488 (38.2)

pDDI, potential drug-drug interaction; MPR, medication possession ratio.

All values are presented as N (%) unless otherwise indicated.

^a^ Received tramadol prescriptions from more than two different medical institutions.

^b^ Concomitant use of potentially inappropriate co-medications ≥ 10 days.

Table 2 shows the characteristics of the entire study population and each latent class. The mean age of the study population was 75.1 years (SD, 6.3), and 68.3% of them were females. In each class, the mean ages were similar (approximately 75 years), whereas the proportion of patients over 85 years was relatively low in antihistamines-tramadol users. Unlike other classes, in which 70% of the patients were females, antihistamines-tramadol users had the lowest proportion of females (59.2%). Anxiolytics-tramadol users were the most likely to have medical aid or veteran health service (14.9%). More than 75% of antidepressants-tramadol users were prescribed tramadol in one medical institution; however, only 43.7% of multiple pDDI combination users received tramadol in one institution. Multiple pDDI combination users had the highest proportion of patients with 3 or more CCI scores (60.5%), followed by antidepressants-tramadol, anxiolytics-tramadol, and antihistamines-tramadol users (Table 2).

**Table 2:** Characteristics of the four latent classes of tramadol users with potential drug-drug interactions.

Characteristics	All	Class 1	Class 2	Class 3	Class 4
		(multiple pDDI combination users)	(antihistamines-tramadol users)	(antidepressants-tramadol users)	(anxiolytics-tramadol users)
**Female sex^a^**	140,427 (68.3)	45,747 (71.7)	32,393 (63.9)	23,156 (69.9)	39,131 (69.5)
**Age^a^^b^**					
**Mean ± SD**	75.05 ± 6.3	75.62 ± 6.16	74.21 ± 6.21	75.2 ± 6.4	75.07 ± 6.39
**65–74**	102,770 (50.4)	29,413 (46.1)	28,554 (56.3)	16,384 (49.5)	28,419 (50.5)
**75–84**	84,663 (41.5)	28,902 (45.3)	18,820 (37.1)	13,826 (41.8)	23,115 (41)
**85+**	16,505 (8.1)	5,512 (8.6)	3,307 (6.5)	2,903 (8.8)	4,783 (8.5)
**Type of insurance^a^^,^^b^**					
**Health insurance**	181,634 (89.1)	54,856 (85.9)	46,245 (91.2)	29,319 (88.5)	51,214 (90.9)
**Others^c^**	22,304 (10.9)	8,971 (14.1)	4,436 (8.8)	3,794 (11.5)	5,103 (9.1)
**Number of visited institutions for tramadol prescription ^a^^,^^b^**					
**Mean ± SD**	1.55 ± 0.85	1.89 ± 1.03	1.4 ± 0.7	1.32 ± 0.65	1.44 ± 0.73
**1**	126,095 (61.8)	27,879 (43.7)	35,108 (69.3)	25,212 (76.1)	37,896 (67.3)
**≥ 2**	77,843 (38.2)	35,948 (56.3)	15,573 (30.7)	7,901 (23.9)	18,421 (32.7)
**MPR of tramadol^b^**					
**Mean ± SD**	88.9 ± 103.7	160.6 ± 120.7	67.2 ± 86.7	57.4 ± 77.24	45.7 ± 58.6
**Median(Q1, Q3)**	41(13, 129)	132(48, 265)	29 (9, 91)	25 (8, 71)	22 (8, 59)
**Duration of concomitant use^b^**					
**Mean ± SD**	55.3 ± 106.1	127.8 ± 154.5	10 ± 19.8	49.3 ± 75.3	17.5 ± 28
**Median(Q1, Q3)**	14 (5, 47)	58 (24, 180)	5 (4, 9)	17 (8, 53)	8 (4, 16)
**Type of institution prescribing tramadol ^a^^,^^d^**					
**General hospital**	36,895 (18.1)	12,218 (19.1)	6,123 (12.1)	9,576 (28.9)	8,978 (15.9)
**Hospital**	23,162 (11.4)	6,858 (10.7)	4,813 (9.5)	5,029 (15.2)	6,462 (11.5)
**Clinic**	143,881 (70.6)	44,751 (70.1)	39,745 (78.4)	18,508 (55.9)	40,877 (72.6)
**Geographic regions^a^**					
**Seoul**	67,120 (32.9)	19,368 (30.3)	16,423 (32.4)	12,053 (36.4)	19,276 (34.2)
**Urban area**	41,522 (20.4)	13,537 (21.2)	10,085 (19.9)	6,987 (21.1)	10,913 (19.4)
**Rural area**	95,296 (46.7)	30,922 (48.4)	24,173 (47.7)	14,073 (42.5)	26,128 (46.4)
**Specialties^a^**					
**Internal Medicine**	118,350 (58)	47,676 (74.7)	29,754 (58.7)	13,680 (41.3)	27,240 (48.4)
**Neurology**	27,863 (13.7)	12,963 (20.3)	1,073 (2.1)	7,134 (21.5)	6,693 (11.9)
**General Surgery**	18,151 (8.9)	7,887 (12.4)	3,660 (7.2)	1,980 (6)	4,624 (8.2)
**Psychiatry**	23,925(11.7)	11,671(18.3)	208(0.4)	8,525(25.7)	3,521(6.3)
**Neurosurgery**	24,657 (12.1)	10,970 (17.2)	2,757 (5.4)	4,332 (13.1)	6,598 (11.7)
**Rehabilitation**	5,496 (2.7)	2,408 (3.8)	650 (1.3)	1,483 (4.5)	955 (1.7)
**Family Medicine**	18,588 (9.1)	8,074 (12.6)	3,995 (7.9)	2,088 (6.3)	4,431 (7.9)
**Comorbidities^a^**					
**Myocardial infarction**	6,775 (3.3)	2,353 (3.7)	1,375 (2.7)	1,206 (3.6)	1,841 (3.3)
**Congestive heart failure**	28,752 (14.1)	10,409 (16.3)	5,591 (11)	5,022 (15.2)	7,730 (13.7)
**Cerebrovascular disease**	49,737 (24.4)	17,621 (27.6)	8,155 (16.1)	10,574 (31.9)	13,387 (23.8)
**Renal disease**	8,518 (4.2)	2,948 (4.6)	1,722 (3.4)	1,718 (5.2)	2,130 (3.8)
**Liver disease**	66,536 (32.6)	23,065 (36.1)	13,858 (27.3)	11,415 (34.5)	18,198 (32.3)
**CCI^b^**					
**Mean ± SD**	3.1 ± 1.8	3.4 ± 1.8	2.7 ± 1.6	3.3 ± 1.8	3.0 ± 1.7
**Median(1Q, 3Q)**	3 (1, 4)	3 (2, 4)	2 (1, 3)	3 (2, 4)	2 (1, 4)

pDDI, potential drug-drug interaction; MPR, medication possession ratio; SD, standard deviation; CCI, Charlson comorbidity index.

^a^P-values (< 0.001) were calculated using the chi-squared test.

^b^P-values (< 0.001) were calculated using the ANOVA test.

^c^Others included Medical aid or veteran health service.

^d^Healthcare institution that mostly prescribed tramadol in 2016.

Table 3 shows the crude and adjusted ORs for potential ADRs. Antihistamines-tramadol users were selected as a reference group. Potential ADR risk was higher in multiple pDDI (adjusted OR, 1.81; 95% CI, 1.75–1.88), antidepressants-tramadol (1.24; 1.19–1.29), and anxiolytics-tramadol users (1.04; 1.00–1.08) than antihistamines-tramadol users.

**Table 3:** Association between latent classes and potential adverse drug reactions.

Definition of potential ADRs	Total	Number of pADRs (%)	OR (95% CI)	Adjusted OR (95% CI)^a^
ED visits				
Class 2: antihistamines-tramadol users	50,681	5,257 (10.4)	1	1
Class 1: multiple pDDI combination users	63,827	12,793 (20)	2.17 (2.09–2.24)	1.81 (1.75–1.88)
Class 3: Antidepressants-tramadol users	33,113	4,967 (15)	1.52 (1.46–1.59)	1.24 (1.19–1.29)
Class 4: anxiolytics-tramadol users	56,317	6,739 (12)	1.17 (1.13–1.22)	1.04 (1.00–1.08)

pADRs: potential adverse drug reactions; ED, emergency department; OR, odds ratio; CI, confidence interval.

^a^Adjusted for age, sex, type of insurance, myocardial infarction, congestive heart failure, cerebrovascular disease, renal disease, liver disease, and geographic regions.

## Discussion

In this population-based LCA study among older adults using tramadol, we identified four distinct groups of potentially inappropriate co-medications with tramadol. Our study showed that the clinical characteristics and occurrence of potential ADRs were significantly different among classes.

Multiple pDDI combination users with the highest prevalence of potential ADRs tended to visit more medical institutions and had a higher CCI score. After adjustment for other risk factors, potential ADR risk was significantly higher in multiple pDDI, antidepressants-tramadol, and anxiolytics-tramadol users than that in antihistamines-tramadol users.

This study showed that the prevalence of potential ADRs was the highest in multiple pDDI combination users, potentially owing to the frequent drug interactions and longer duration of concomitant use. It seems that the longer duration of tramadol use observed in the multiple pDDI group probably had an impact on the likelihood of being co-administration with various inappropriate drugs rather than a characteristic after becoming multiple pDDI groups. According to the cohort study of outpatient pediatric polypharmacy showed similar patterns that depth of polypharmacy was correlated with the number of total prescriptions [24]. A retrospective cohort study of older patients in the United States showed that polypharmacy increased ADR-related hospitalization risk after adjusting for confounding factors, including demographic and health-status control variables [11]. Calderón-Larrañaga et al. reported that not only polypharmacy but also the number of physician visits and number of different specialties were risk factors for adverse drug events [25]. These results are in line with our findings that multiple pDDI combination users tended to visit multiple medical institutions more frequently than the other groups did. Besides interaction with tramadol, interaction between the co-medications could increase risk of CNS adverse effects. Beers criteria, the most widely used criteria for potentially inappropriate co-medications in older people, recommended avoiding the concomitant use of anticholinergics, including first-generation antihistamines and TCAs [26]. In addition, the Screening Tool of Older Persons’ Prescriptions (STOPP) stated that simultaneous use of two or more drugs with anticholinergic effects could increase the anticholinergic toxicity [27].

Among the groups receiving only one potentially inappropriate co-medication, the prevalence of potential ADRs was relatively high in antidepressants-tramadol users. The long duration of antidepressants use required for the pharmacological treatment of depression [28, 29] might explain the relatively long duration of concomitant use observed in antidepressants-tramadol users. The antidepressants-tramadol group had the about 50% of probability to the use of anxiolytics, which could imply that this group had a relatively severe psychiatric condition, and it seems that antidepressants-tramadol users likely to receive a tramadol prescription in hospital or by the psychiatry department.

Furthermore, Onder et al. reported that depression itself might increase ADR risk, particularly in women [30]. The high prevalence of potential ADRs among antidepressants-tramadol users might be attributed to the high proportion of females in this group and the association between depression and ADR risk. When the frequency of each antidepressant use was assessed, most of the patients were found to receive SSRIs or TCA, whereas very few patients were prescribed MAOIs. Since concomitant MAOI and tramadol use is contraindicated according to the Korean nationwide drug utilization review program, their concomitant use was reduced by pop-up alert providing safety information [31]. Because the use of tramadol with antidepressants is increasing, well-designed studies assessing the risk of concomitant use of tramadol and antidepressants, providing evidence that appropriate use of both drugs might be needed [2, 32].

Although first-generation antihistamines and anxiolytics were considered potentially inappropriate medications in older patients [27], the prevalence of potential ADRs was relatively low. Most of the patients concomitantly used these two drugs on temporary basis. Antihistamines-tramadol users who showed the lowest prevalence of potential ADRs exhibited few risk factors for potential ADRs, such as low percentage of older patients aged 85 years or older and less comorbidity. Unlike other groups, the proportion of males was relatively high among antihistamines-tramadol users, and this sex difference might contribute to the low prevalence of potential ADRs in this group [33, 34]. Although the prevalence of potential ADRs among tramadol-antihistamines users was low, appropriate management was needed because of the anticholinergic effects and SS reported in previous studies [10, 16]. In particular, acute ADRs might occur in tramadol-antihistamines users if no sufficient attention was given to ADRs because patients were relatively younger, frequently visited the clinic, or had less comorbidity and a relatively short duration of concomitant use.

Anxiolytics-tramadol users included relatively more medical aid beneficiaries, whose income was less than the legal minimum cost of living. According to a previous study conducted using the Korean national health database, medical aid population exhibited increased polypharmacy risk [35]. Besides respiratory depression and overdose risk in concurrent users of anxiolytics and tramadol, ADR-related factors, including polypharmacy, should be considered in anxiolytics-tramadol users.

Significant differences in potential ADR risk observed among the four classes after adjustment for other risk factors should be cautiously interpreted because our LCA model was constructed based on various patient characteristics and healthcare utilization patterns; thus, the differences were not solely associated with each potentially inappropriate co-medication (antidepressants or anxiolytics), compared with antihistamines.

To our knowledge, this is the first study to classify tramadol users with potentially inappropriate co-medications in older adults using LCA. The generalizability of our findings is ensured because we used national health insurance database covering nearly the entire Korean population. The use of a computerized database minimized the possibility of recall bias for drug use. Additionally, the overall use of tramadol and potentially inappropriate co-medications was thoroughly examined because all study drugs were prescription drugs.

However, this study had some limitations. Although we defined potential ADRs as all-cause ED visits, which may also be associated with patients’ underlying diseases or conditions; however, the probability of misclassification would be comparable between the distinct latent classes. Additionally, we could not confirm whether the drugs were administered as prescribed. The definition of healthcare switching did not take into account the order of outpatient visits and hospitalizations that may affect the appropriateness. However, the results of LCA grouping and association between latent classes and potential ADRs were similar to the main results, according to the sensitivity analysis among patients excepting hospitalized at least once in 2016. Due to the limitation of study design, interpretability of characterizations, and limited time period of database, no further examination was conducted considering mortality and the drug utilization pattern (new-user or prevalent user) of tramadol. Our analysis included only first generation antihistamine, antidepressants and anxiolytics as the potential DDI in the latent class, to consider mainly central nervous system adverse reactions. We suggest that further studies are conducted to evaluate the associations between latent classes and ADRs in tramadol users, which take into account the effect of death, dose of study drugs, and the detailed utilization pattern of tramadol and various co-medications.

## Conclusions

In our study, four distinct classes were identified among older adults using tramadol and potentially inappropriate co-medications, and differences in potential ADR risk was observed between classes. Although tramadol is an effective weak opioid analgesic, careful monitoring of potential central nervous system adverse reactions in older adults is needed, especially when used with concomitant medications which may trigger the adverse effects. Furthermore, when antidepressants or anxiolytics treatment is necessary in an elderly patient with chronic pain, using alternative analgesics such as acetaminophen instead of tramadol needs to be considered.

## Supporting information

S1 Table List of potentially interacting drugs with tramadol. (DOCX)

S2 Table Latent class analysis model fit statistics. AIC, Akaike information criterion; BIC, Bayesian information criterion; G^2^, G^2^ statistic; X^2^, chi-squared statistic.
(DOCX)

S3 Table Association between latent classes and potential adverse drug reactions stratified by sex. pADRs: potential adverse drug reactions; ED, emergency department; OR, odds ratio; CI, confidence interval. ^a^Adjusted for age, sex, type of insurance, myocardial infarction, congestive heart failure, cerebrovascular disease, renal disease, liver disease and geographic regions.
(DOCX)

S1 Fig **A.** Class probability in the four latent classes of male tramadol users with potential drug-drug interactions. **B.** Class probability in the four latent classes of female tramadol users with potential drug-drug interactions.
(DOCX)
