# Supplementary material for: Characterizing tramadol users with potentially inappropriate co-medications: A latent class analysis among older adults
Source: PLoS One. 2021 Feb 19;16(2):e0246426. doi: 10.1371/journal.pone.0246426 (PMC7894862; doi:10.1371/journal.pone.0246426)
Supplement: S1 Table — (DOCX) [file pone.0246426.s001.docx]

S1 Table. List of potentially interacting drugs with tramadol

| Therapeutic class | | Medication |
| --- | --- | --- |
| Antidepressants | |  |
|  | Serotonin reuptake inhibitors | fluoxetine, citalopram, paroxetine, sertraline, fluvoxamine, escitalopram, mianserin, trazodone, nefazodone, mirtazapine, venlafaxine, milnacipran, duloxetine, and desvenlafaxine |
|  | Tricyclic antidepressants | imipramine, clomipramine, amitriptyline, nortriptyline, doxepin, amoxapine, maprotiline, quinupramine, and tianeptine |
|  | Monoamine oxidase inhibitors | moclobemide and minaprine |
|  | Other antidepressants | bupropion, medifoxamine, agomelatine, Hyperici herba, and vortioxetine |
| First-generation antihistamines | | diphenhydramine, clemastine, diphenylpyraline, carbinoxamine, doxylamine, brompheniramine,  chlorphenamine, pheniramine, promethazine, mequitazine, buclizine, oxatomide, cyproheptadine, phenindamine, triprolidine, azatadine, and homochlorcyclizine |
| Anxiolytics | |  |
|  | Benzodiazepines | clonazepam, diazepam, chlordiazepoxide, oxazepam, clorazepate, lorazepam, bromazepam, clobazam, alprazolam, pinazepam, nordazepam, fludiazepam, loflazepate, etizolam, clotiazepam, tofisopam, flurazepam, flunitrazepam, estazolam, triazolam, lormetazepam, temazepam, midazolam, brotizolam, quazepam, loprazolam, and flutoprazepam |
|  | Non-benzodiazepines | zopiclone and zolpidem |
