# Supplementary material for: Characterizing tramadol users with potentially inappropriate co-medications: A latent class analysis among older adults
Source: PLoS One. 2021 Feb 19;16(2):e0246426. doi: 10.1371/journal.pone.0246426 (PMC7894862; doi:10.1371/journal.pone.0246426)
Supplement: S2 Table — AIC, Akaike information criterion; BIC, Bayesian information criterion; G2, G2 statistic; X2, chi-squared statistic. (DOCX) [file pone.0246426.s002.docx]

S2 Table. Latent class analysis model fit statistics

| Number of class | AIC | BIC | G^2^ | X^2^ |
| --- | --- | --- | --- | --- |
| 2 | 1,709,164 | 1,709,318 | 98,258 | 105,091 |
| 3 | 1,664,231 | 1,664,466 | 53,308 | 52,156 |
| 4 | 1,627,331 | 1,627,648 | 16,393 | 15,695 |
| 5 | 1,622,599 | 1,622,997 | 11,644 | 11,354 |
| 6 | 1,618,092 | 1,618,572 | 7,121 | 6,715 |

AIC, Akaike information criterion; BIC, Bayesian information criterion; G^2^, G^2^ statistic; X^2^, chi-squared statistic
