# Supplementary material for: Characterizing tramadol users with potentially inappropriate co-medications: A latent class analysis among older adults
Source: PLoS One. 2021 Feb 19;16(2):e0246426. doi: 10.1371/journal.pone.0246426 (PMC7894862; doi:10.1371/journal.pone.0246426)
Supplement: S3 Table — pADRs: potential adverse drug reactions; ED, emergency department; OR, odds ratio; CI, confidence interval. aAdjusted for age, sex, type of insurance, myocardial infarction, congestive heart failure, cerebrovascular disease, renal disease, liver disease and geographic regions. (DOCX) [file pone.0246426.s003.docx]

**S3 Table. Association between latent classes and potential adverse drug reactions stratified by sex**

| Definition of potential ADRs | Total | Number of pADRs (%) | OR (95% CI) | Adjusted OR  (95% CI)^a^ |
| --- | --- | --- | --- | --- |
| Male (n=63,511) |  |  |  |  |
| Class 2: antihistamines-tramadol users | 18,317 | 2,107 | 1 | 1 |
| Class 1: multiple pDDI combination users | 16,671 | 3,671 | 2.17 (2.05-2.30) | 1.75 (1.64-1.86) |
| Class 3: Antidepressants-tramadol users | 11,300 | 1,922 | 1.58 (1.47-1.69) | 1.25 (1.17-1.34) |
| Class 4: anxiolytics-tramadol users | 17,223 | 2,203 | 1.13 (1.06-1.20) | 0.98 (0.91-1.04) |
| Female (n=140,427) |  |  |  |  |
| Class 2: antihistamines-tramadol users | 32,466 | 3,172 | 1 | 1 |
| Class 1: multiple pDDI combination users | 45,468 | 8,750 | 2.20 (2.11-2.30) | 1.85 (1.77-1.93) |
| Class 3: Antidepressants-tramadol users | 23,247 | 3,374 | 1.57 (1.49-1.65) | 1.27 (1.21-1.34) |
| Class 4: anxiolytics-tramadol users | 39,246 | 4,557 | 1.21 (1.16-1.27) | 1.07 (1.02-1.12) |

pADRs: potential adverse drug reactions; ED, emergency department; OR, odds ratio; CI, confidence interval

^a^Adjusted for age, sex, type of insurance, myocardial infarction, congestive heart failure, cerebrovascular disease, renal disease, liver disease and geographic regions.
