# Supplementary material for: Characterizing tramadol users with potentially inappropriate co-medications: A latent class analysis among older adults
Source: PLoS One. 2021 Feb 19;16(2):e0246426. doi: 10.1371/journal.pone.0246426 (PMC7894862; doi:10.1371/journal.pone.0246426)
Supplement: S1 Fig — A. Class probability in the four latent classes of male tramadol users with potential drug-drug interactions. B. Class probability in the four latent classes of female tramadol users with potential drug-drug interactions. (DOCX) [file pone.0246426.s004.docx]

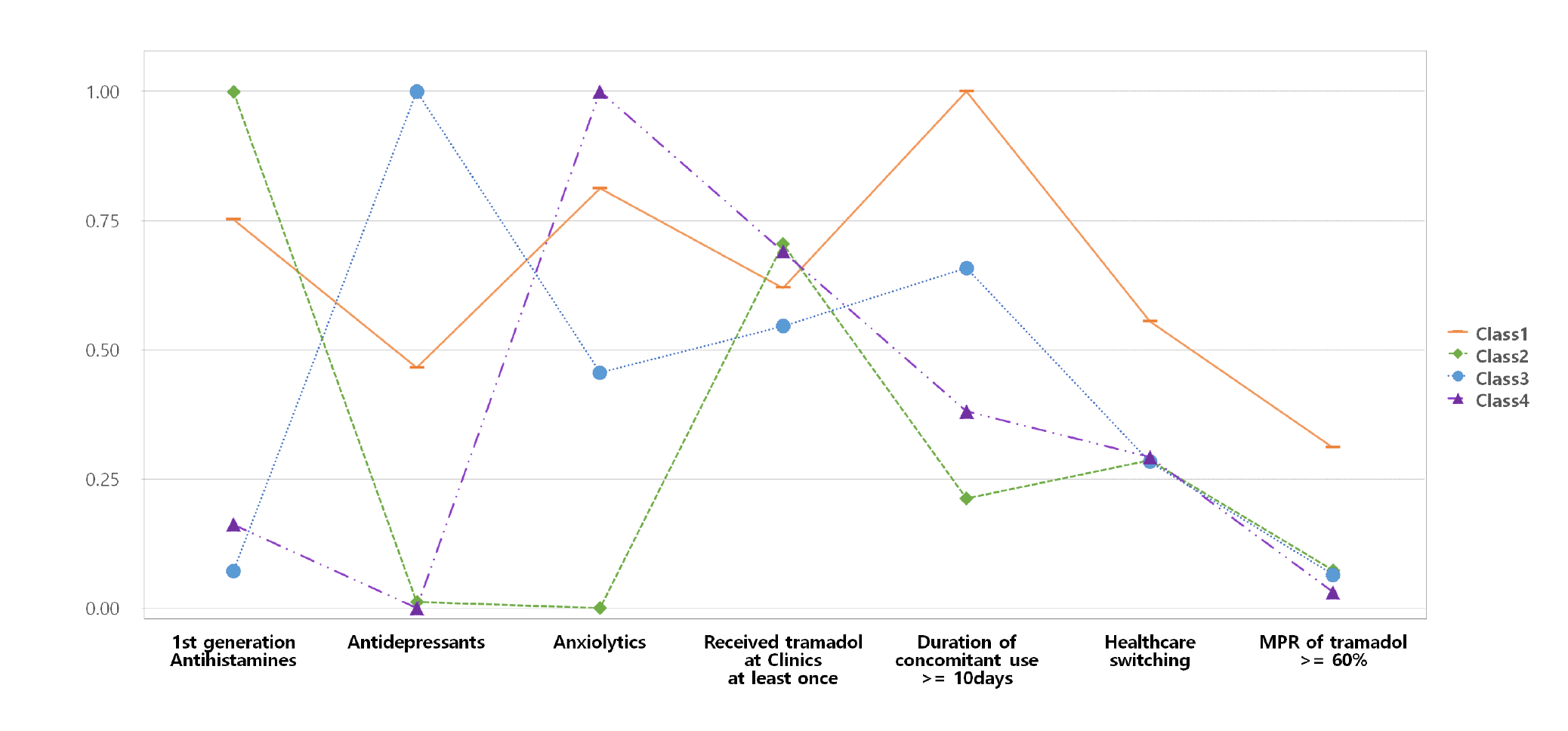


**S1A Fig. Class probability in the four latent classes of male tramadol users with potential drug-drug interactions**


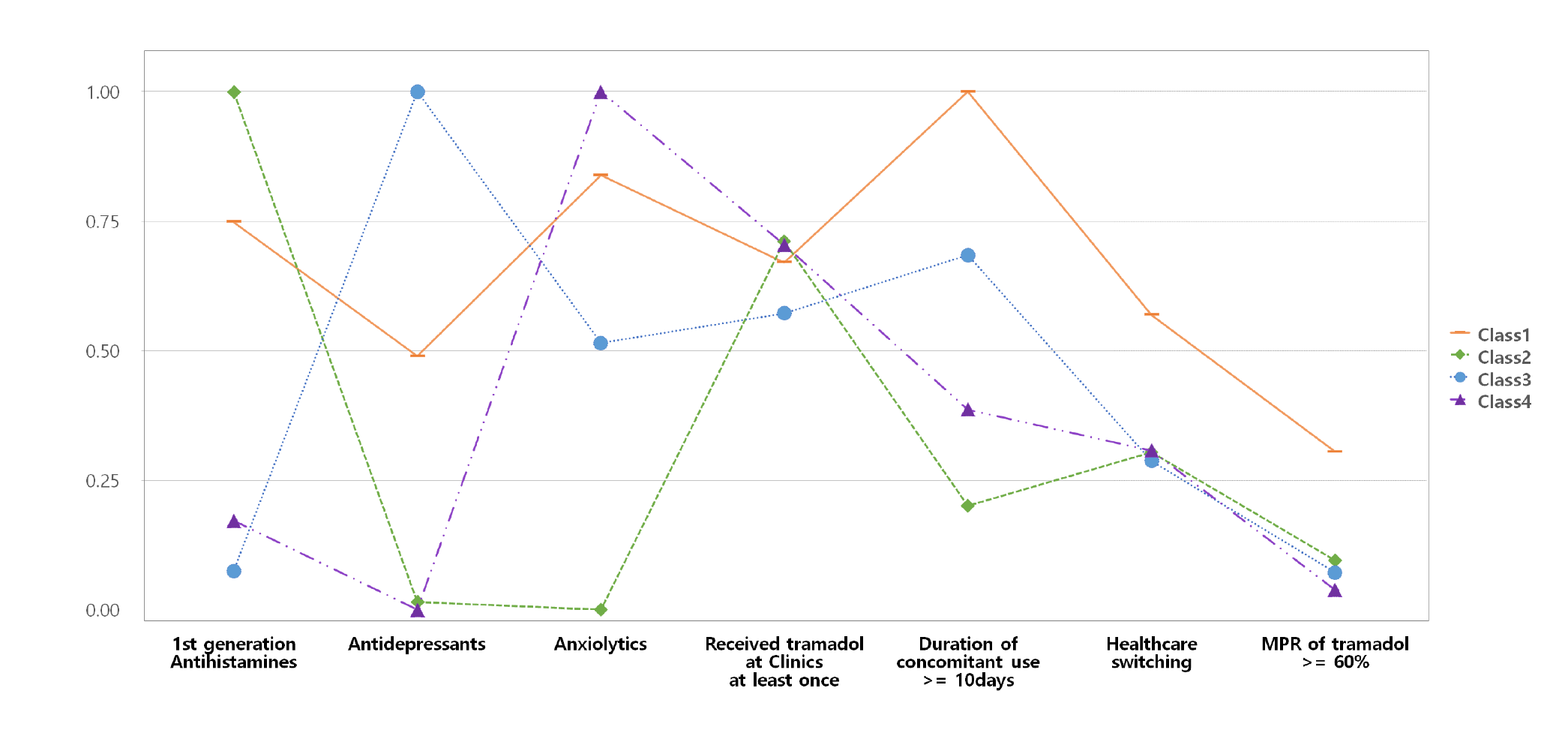
**S1B Fig. Class probability in the four latent classes of female tramadol users with potential drug-drug interactions**
